# Prurigo Pigmentosa Induced by a Ketogenic Diet

**DOI:** 10.7759/cureus.39498

**Published:** 2023-05-25

**Authors:** Aditya Nellore, Eamonn Maher, Mallory Abate

**Affiliations:** 1 Dermatology, Saint Louis University School of Medicine, Saint Louis, USA; 2 Internal Medicine, Saint Luke's Hospital, Chesterfield, USA; 3 Dermatology, University of Minnesota Medical School, Minneapolis, USA; 4 Dermatology, The Dermatology Clinic, Baton Rouge, USA

**Keywords:** skin, ketogenic diet, ketosis, dermatology, prurigo pigmentosa

## Abstract

Prurigo pigmentosa is an important cause of reticular pruritic rash that has been under-reported in the United States. To ensure proper patient care, it is important for dermatologists to be aware of its presentations and associated factors. Here, we present an uncommon case of prurigo pigmentosa induced by a ketogenic diet and discuss the links between this condition and the state of ketosis, a relationship every provider should be conscious of.

## Introduction

Prurigo pigmentosa (PP) is a reticulated pigmentary disorder first described by Nagashima in 1971 [1]. The majority of cases are seen in Japanese women, with a mean age of onset between 23 and 27 years [2]. However, while rare, there have been reports of men, children, and individuals of other ethnicities afflicted with this illness [3-7]. This condition has been somewhat under-reported in the United States but is important for dermatologists to be aware of to ensure effective patient care [8].

The pathogenesis of PP has been associated with a multitude of factors, such as systemic conditions, hormonal triggers, and dietary changes [9]. Particularly, there is a growing association between ketosis and the development of PP, indicating that any factor inducing this metabolic state may trigger PP [4,9,10]. This link is important for dermatologists to be aware of, as the ketogenic diet has exploded in popularity in recent years. This diet involves substituting dietary carbohydrates with protein and fat, essentially forcing the body into ketosis, with subsequent rapid weight loss. Cases of PP induced by a ketogenic diet can often be treated with diet change alone, minimizing the need to expose patients to unnecessary medications and the side effects associated with them. Here, we present a case of PP in a 26-year-old Caucasian American woman who had recently begun a ketogenic diet.

## Case presentation

**Figure 1:**
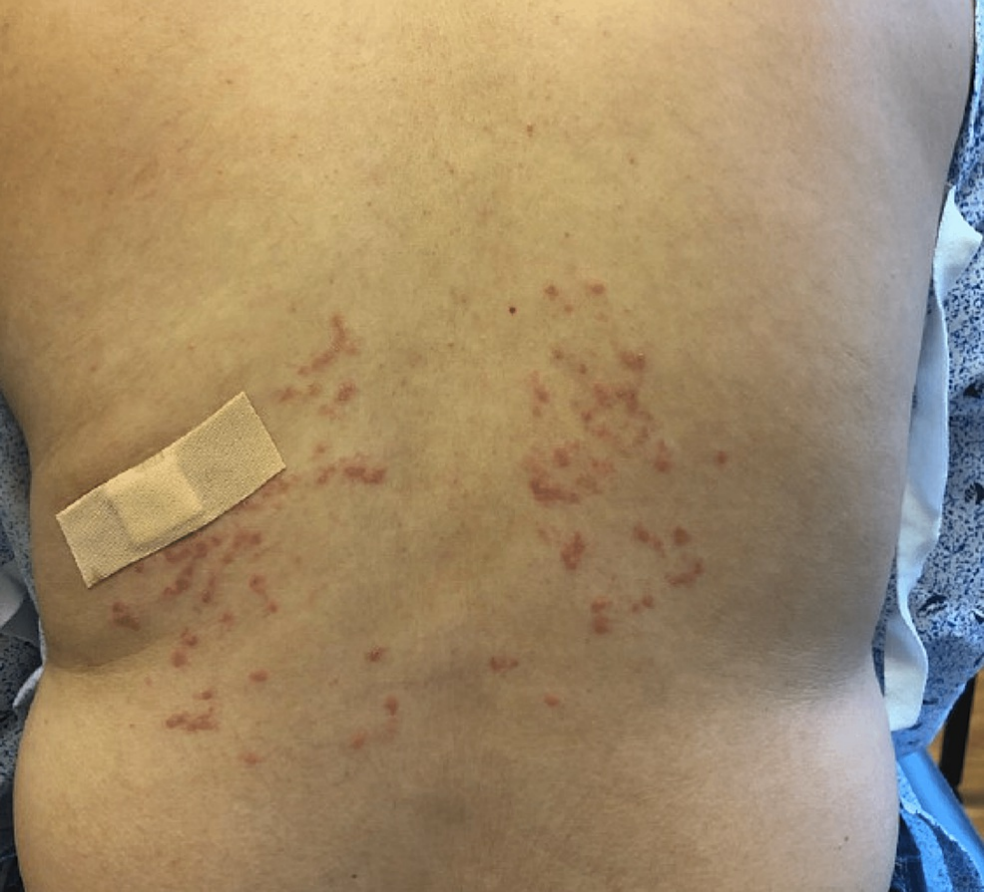
Prurigo pigmentosa. Numerous reticulated pink papules with mild scale on the mid-back.

An otherwise healthy 26-year-old woman presented with a three-week history of pruritic rash located on the middle back, upper chest, and supraclavicular area. Examination revealed numerous reticulated pink papules with mild scale (Figure 1).

**Figure 2:**
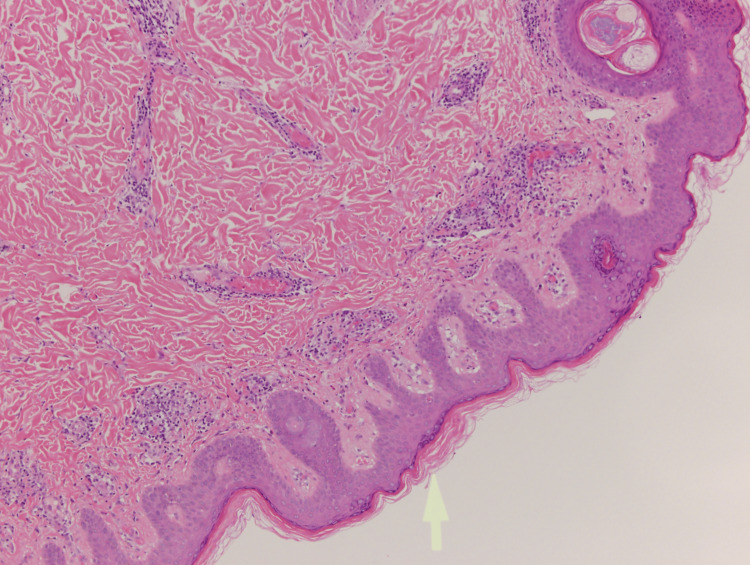
Punch biopsy of lesions on the patient’s back. Focal parakeratosis, scattered dyskeratotic keratinocytes, and melanophages around the superficial vascular plexus (H&E, 4×).

**Figure 3:**
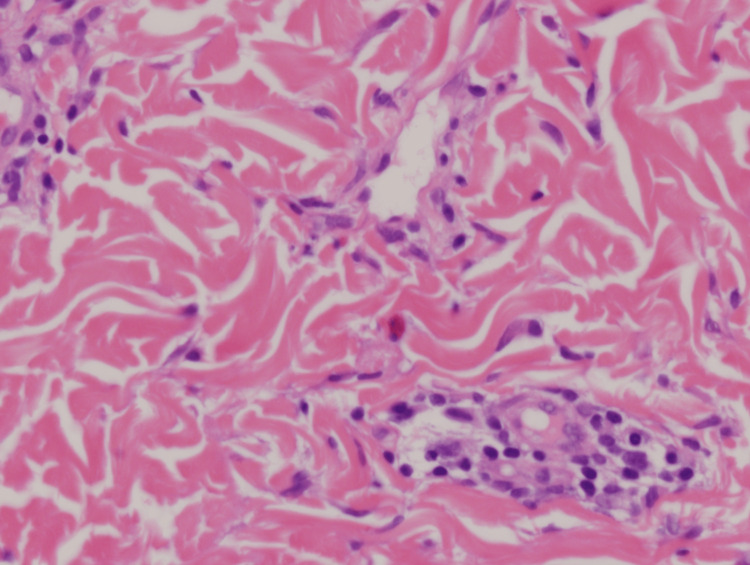
Mid-dermal perivascular lymphocytic infiltrate with scattered eosinophils (H&E, 400×).

The patient began a strict ketogenic diet three weeks prior to rash onset, during that time she experienced successful weight loss with no additional side effects. The review of systems was negative. The patient was not exposed to any other possible causative factors. Punch biopsy was taken, which demonstrated epidermal focal parakeratosis and scattered dyskeratotic keratinocytes, superficial and mid-dermal perivascular lymphocytic infiltrate with scattered eosinophils and neutrophils, and abundant melanin within melanophages around the superficial vascular plexus (Figures 2, 3).

**Figure 4:**
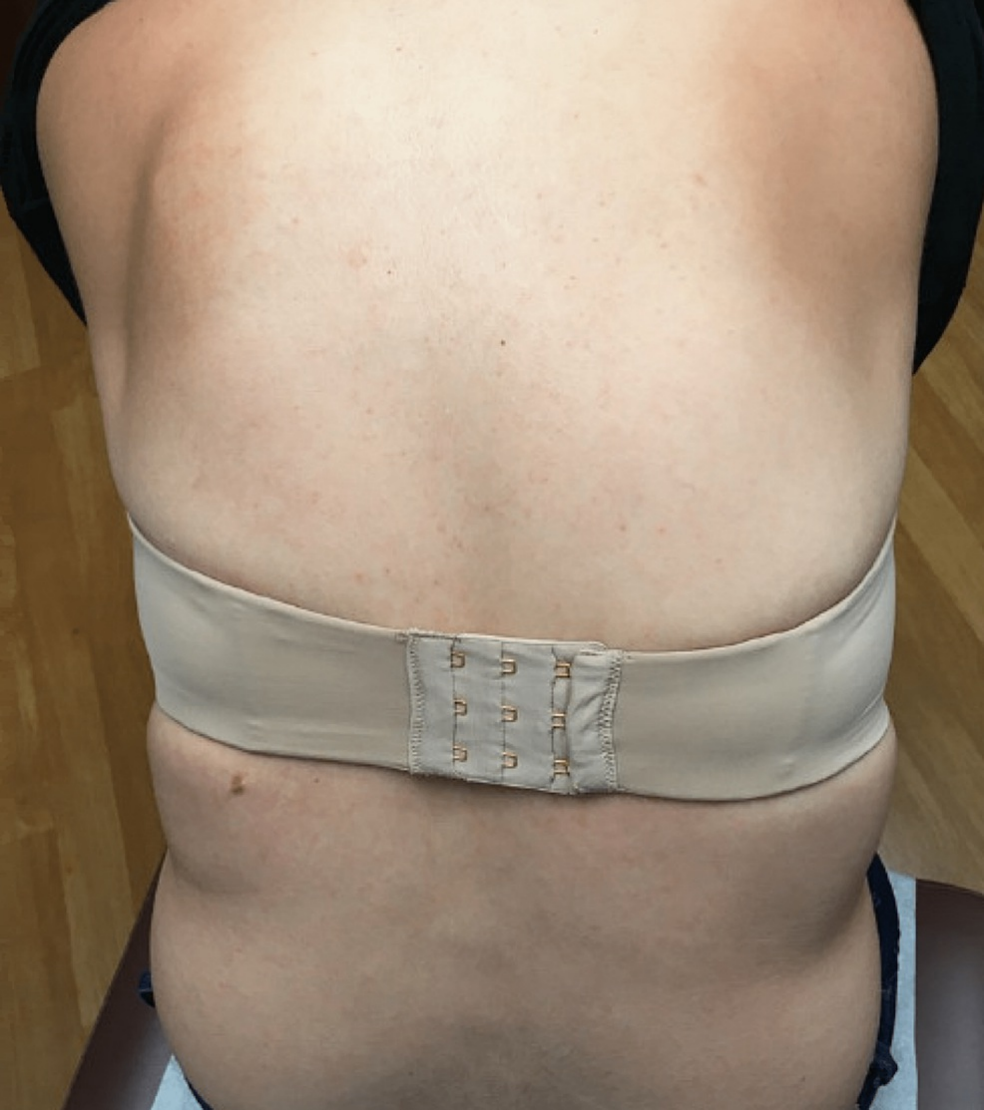
Patient’s mid-back one month after reintroducing carbohydrates into her diet.

Based on the characteristics of her lesions and their appearance following the onset of a ketogenic diet, in addition to the aforementioned supportive histologic findings, a diagnosis of prurigo pigmentosa secondary to ketosis was made. The patient’s rash resolved within one month of reintroducing carbohydrates into her diet, with no other treatment (Figure 4).

## Discussion

Prurigo pigmentosa initially presents with intensely pruritic erythematous papules, typically on the neck, chest, back, and rarely, the limbs or face. There are no reports of the condition affecting mucous membranes or nails [8]. These lesions develop rapidly and then regress within a week, leaving behind a reticulated hyperpigmentation [6]. The early-stage papules can resemble a host of other conditions, such as dermatitis herpetiformis, impetigo, erythema multiforme, and linear IgA disease [11,12]. The late-stage reticulated pigmentation, on the other hand, can mimic nonspecific forms of postinflammatory hyperpigmentation.

Each of these stages can be characterized histologically. The pruritic lesions initially display a superficial perivascular neutrophilic infiltrate within the epidermis and papillary dermis. This rapidly progresses to spongiosis, necrotic keratinocytes, and a lymphocytic infiltrate in the upper dermis [5,9,13]. Once the lesions become hyperpigmented, melanophages become visible in the upper dermis, with the epidermis appearing hyperplastic and parakeratotic [13]. Treatment for PP typically involves antibiotics, such as dapsone or minocycline, but can be treated with diet changes in ketosis-induced cases [9].

Despite having an understanding of the clinical and histopathological features of PP, the pathogenesis of the condition is still unclear. However, several hypotheses have been proposed. Systemic conditions may contribute, such as atopy, *Helicobacter pylori *infection, and Sjogren’s syndrome [7,14,15]. Prurigo pigmentosa has also been shown to arise during periods of hormonal change, such as during pregnancy [16]. Lastly, as previously mentioned, there has been shown to be an association between PP and metabolic or diet changes, most importantly the state of ketosis in the body. While this relationship is not totally understood, it is theorized that ketone bodies can induce neutrophil-mediated perivascular inflammation, a theory supported by the fact that PP responds well to medications that inhibit neutrophils, such as tetracyclines or dapsone [17].

With this in mind, it stands to reason that any change resulting in ketonemia could cause a PP eruption. Such changes include diabetes mellitus, obesity, bariatric surgery, anorexia nervosa, fasting, and strict dieting, all of which have been reported as likely causes of PP cases around the world [3-4,18,19]. All of these aside, perhaps the most direct way to induce ketosis in the body is through a ketogenic diet, which consists of drastically reducing carbohydrate intake and replacing it with fat and protein. This, in turn, forces the body to turn fat into ketone bodies that can be used for energy. Cases of PP induced, seemingly solely, by a ketogenic diet have been reported, many of which experienced resolution of symptoms with antibiotics [4,10]. However, antibiotics have been shown to successfully treat PP induced by any cause, not just by a ketogenic diet. Our case is one of the few reported cases that was treated exclusively with the reintroduction of carbohydrates into the diet, which is highly suggestive of our patient’s condition being brought about by her ketogenic diet alone [9].

## Conclusions

With the ketogenic diet growing in popularity, it is important for physicians to be aware of the relationship between PP and ketosis. When treating patients with reticular pruritic rashes on the trunk, providers should always screen for a ketogenic diet - the reintroduction of carbohydrates into the diet may be sufficient to treat such patients, eliminating the need to prescribe medication that could result in unpleasant side effects.
